# Stacking Machine Learning Algorithms for Biomarker-Based Preoperative Diagnosis of a Pelvic Mass

**DOI:** 10.3390/cancers14051291

**Published:** 2022-03-02

**Authors:** Reid Shaw, Anna E. Lokshin, Michael C. Miller, Geralyn Messerlian-Lambert, Richard G. Moore

**Affiliations:** 1 Division of Gynecologic Oncology, Department of Obstetrics and Gynecology, Wilmot Cancer Institute, University of Rochester, Rochester, NY 14642, USA; reid.shaw@luhs.org (R.S.); usmc_aggie90@yahoo.com (M.C.M.); 2 Hillman Cancer Center, University of Pittsburgh, Pittsburg, PA 15219, USA; loksax@upmc.edu; 3 Department of Pathology, Women and Infants Hospital/Brown University, Providence, RI 02905, USA; gmesserlian@wihri.org

**Keywords:** pelvic mass, HE4, CA125, ovarian cancer

## Abstract

**Simple Summary:**

It is critical for women who are diagnosed with a pelvic mass, or an ovarian cyst to be accurately assessed for their risk of having an ovarian malignancy. Accurate risk stratification for these women will allow for appropriate triage and referral to centers best equipped to treat women diagnosed with ovarian cancer. In this study, machine learning (ML) algorithms were used to determine the optimal combination of biomarkers for prediction of malignancy in women presenting with a pelvic mass. Nine unique ML algorithms were employed to evaluate age, menopausal status, race, and levels of 67 biomarkers from serum, urine, and plasma samples prospectively collected in a cohort 140 women with a variety of pelvic mass diagnoses benign and malignant. A complex statistical algorithm using serum levels of CA125, HE4 and transferrin provided greater than 93% sensitivity and specificity for the preoperative prediction of malignancy in women presenting with a pelvic mass.

**Abstract:**

Objective: To identify the most predictive parameters of ovarian malignancy and develop a machine learning (ML) based algorithm to preoperatively distinguish between a benign and malignant pelvic mass. Methods: Retrospective study of 70 predictive parameters collected from 140 women with a pelvic mass. The women were split into a 3:1 “training” to “testing” dataset. Feature selection was performed using Gini impurity through an embedded random forest model and principal component analysis. Nine unique ML classifiers were assessed across a variety of model-specific hyperparameters using 25 bootstrap resamples of the training data. Model predictions were then combined into an ensemble stack by LASSO regression. The final ensemble stack and individual classifiers were then applied to the testing dataset to assess model performance. Results: Feature selection identified HE4, CA125, and transferrin as three predictive parameters of malignancy. Assessment of the ensemble stack on the testing dataset outperformed all individual ML classifiers in predicting malignancy. The ensemble stack demonstrated an accuracy of 97.1%, a receiver operating characteristic (ROC) area under the curve (AUC) of 0.951, and a sensitivity of 93.3% with a specificity of 100%. Conclusions: Combining the measurement of three distinct biomarkers with the stacking of multiple ML classifiers into an ensemble can provide valuable preoperative diagnostic predictions for patients with a pelvic mass.

## 1. Introduction

Accurate and early identification of a malignant pelvic mass is essential for appropriate triage, referral, and subsequent care for women diagnosed with an ovarian malignancy. Referral of women with a potential malignant pelvic mass or a diagnosis of ovarian cancer to gynecologic oncologists and high-volume institutions has been demonstrated to improve patient outcomes [1,2,3]. Approximately 289,000 women in the United States are diagnosed with a pelvic mass each year, of which 10–20% will subsequently be diagnosed with a malignancy [4]. While most malignant pelvic masses fall into the category of epithelial ovarian cancer (EOC), which includes fallopian tube, ovarian and primary peritoneal malignancies, a small proportion of malignant pelvic masses include other malignancies, such as non-EOC (germ cell and sex cord stromal tumors), endometrial and cervical malignancies, and metastatic disease to the ovaries [5]. Although length of survival following diagnosis has improved dramatically with the current standard of care, overall survival remains poor, as nearly 80% of women diagnosed with fallopian tube and ovarian cancer are identified at an advanced stage [5]. 

Multiple algorithms have been developed to assist physicians with risk stratification of woman diagnosed with an ovarian cyst or pelvic mass. The most common tools use different combinations of biomarkers, imaging, and patient characteristics. Early assessment tools solely used serum measurements of the cancer antigen 125 (CA125) biomarker to stratify patients into high- and low-risk groups for being diagnosed with an EOC. However, questions regarding the specificity of CA125 led to the development of the Risk of Malignancy Index (RMI), which added the patient’s menopausal status and an ultrasound score [6]. While initial results were promising, the variation of ultrasound image quality can cause a significant decrease in algorithm performance [7]. 

More recently, multiple biomarker algorithms have been developed and cleared by the USFDA and European Union for clinical use. The risk of ovarian malignancy algorithm (ROMA^®^) is a logistic regression algorithm that uses serum human epididymis protein 4 (HE4) and CA125 levels along with the patient’s menopausal status to categorize patients into high- and low-risk probabilities that a malignancy will be found in a patient with a pelvic mass [8,9,10]. ROMA has achieved a sensitivity of 94% and a negative predictive value (NPV) of 99% at a set specificity of 75% for predicting the presence of epithelial ovarian cancer in women presenting with a pelvic mass [8,9,10]. An additional risk assessment tool is the multivariate index assay, OVA1^®^. This unpublished algorithm employs menopausal status, serum levels of CA125, transferrin (TRF), transthyretin (prealbumin), apolipoprotein A1 (APOA1), and beta-2-microglobulin to stratify women into high- and low-risk categories [11]. The multivariate index assay demonstrated a sensitivity of 92.4% and a NPV of 96.8% with a specificity of 53.5% [11]. In 2016, a second-generation multivariate index assay, OVERA^®^, was developed. This unpublished algorithm utilizes CA125, HE4, TRF, APOA1, and follicle stimulating hormone (FSH) levels to predict risk of malignancy [12]. This refined algorithm improved upon model specificity while retaining comparable levels of sensitivity—demonstrating a sensitivity of 91.3% with a NPV of 97.2% with a specificity of 69.1% [12]. Although these models significantly improve the ability to detect early-stage disease, there is room for improvement.

Active areas of ovarian cancer research focus on the identification of new markers to detect disease at earlier stages [13]. Additionally, the use of high-throughput technology has identified many additional biomarkers associated with ovarian cancer [14]. The objective of this study was to utilize machine learning (ML) algorithms to determine the optimal combination of biomarkers to predict malignancy in women presenting with a pelvic mass. If a sufficiently predictive model can be built, then patients with malignancy could more efficiently and appropriately be referred to gynecologic oncologists and centers specializing in the care for women diagnosed with a malignancy.

## 2. Materials and Methods

### 2.1. Clinical Patient Evaluation and Measurement of Biomarkers

This study was reviewed by the Institutional Review Board (RSRB office study ID: STUDY00005753), determining that the study meets federal and university criteria for exemption. All data in the data repositories that were used in the current study had previously been de-identified. Inclusion criteria for this trial required all patients have imaging documenting a pelvic mass or ovarian cyst thirty days prior to surgery. All patients had to have a surgery with pathologic documentation of the pelvic mass or ovarian cyst. Each patient had pre-operative levels of HE4, CA125, and other biomarkers drawn prior to surgery. Patients with a prior history of a bilateral salpingoophorectomy were ineligible for the trial. Pregnant patients and women under the age of 18 were excluded from the trial. For all patients in this study a pelvic mass was confirmed by imaging (ultrasound, computed tomography, or magnetic resonance imaging). All patients underwent surgery for a subsequent tumor histological evaluation and full surgical staging for women diagnosed with a malignancy. Malignant tumors were graded and staged in accordance with FIGO classification. Age, menopausal status, race, and 67 biomarkers from serum, urine, and plasma samples were preoperatively collected for each patient (Table S1). The biomarkers were chosen based on previous studies. Serum CA125 and HE4 levels were measured on the ARCHITECT i2000 (Abbot Diagnostics Inc., Chicago, IL, USA), serum YKL-40 levels were measured by MicroVue YKL-40 EIA kit (Quidel Inc., San Diego, CA, USA), serum transthyretin levels were measured by TTR ELISA kit (Cusabio Biotech Co., Houston, TX, USA), serum APOA1 levels were measured by Human apoA1 ELISA kit (Mabtech Inc., Cincinnati, OH, USA), serum beta 2-microglobulin levels were measured by Human beta 2-Microglobin ELISA kit (R&D Systems, Minneapolis, MN, USA), and serum TRF levels were measured on the Advia Chemistry system (Siemens Diagnostics, Tarrytown, NY, USA). The remaining protein biomarkers were analyzed using multiplex bead-based immunoassay on the Luminex platform (Luminex Inc., Austin, TX, USA), as previously described [15]. Kits were purchased from MilliporeSigma (Burlington, MA, USA) Sigma/EMD/Millipore. All proteins were analyzed with the Bio-Plex 200 reader. Standard curves were generated using 5-parameter curve fitting.

### 2.2. Univariate Statistical Analysis and Logistic Regression

Descriptive statistics were calculated for each subject group. Statistical significance of differences for each biomarker was analyzed using the non-parametric Mann–Whitney test. Correlations between results for pairs of assays were assessed by the non-parametric Spearman rank correlation test. Multiple logistic regression (LR) models were fit to the bootstrapped training data. All statistically significant univariates were included in the generation of LR models. LR models were generated by sequentially excluding each biomarker from the formula. 

### 2.3. Data Splitting and Pre-Processing

The 140 patients were pseudo-randomly split into a 3:1 training (*n* = 106) to testing (*n* = 34) dataset and stratified to maintain the proportion of malignant tumors in the complete dataset (Table 1A and Table S2). The training dataset was then bootstrap resampled 25 times for robust model assessment. All models were blinded to the testing data throughout training until the final model was evaluated. Missing values were imputed with K-nearest-neighbors, and menopausal status was one-hot encoded into a binary variable. For models that require normalized data, non-normal distributed continuous predictors were transformed using a Box-Cox transformation. The histologic categorization of malignant tumor type and grade of tumor are displayed in Table 1B,C, respectively.

### 2.4. Feature Selection

To select the most valuable predictors of malignancy, we performed principal component analysis (PCA), and fit an embedded random forest (RF) model using all predictive parameters. These orthogonal approaches assessed 70 unique predictive parameters. To identify the optimal hyperparameters for the RF model, we applied Bayesian optimization, using the expected improvement as the acquisition function [16]. A total of 55 unique hyperparameter combinations were fit to the RF model across each of the 25 bootstrap resamples within the training dataset. The hyperparameters that provided the maximal ROC AUC was used for 1000 subsequent pseudo-random RF model fittings. Gini impurity, the probability of misclassifying an observation, was calculated for each predictive parameter across each model fit. 

### 2.5. Supervised Machine Learning and Ensemble Stacking

We assessed nine different supervised ML classifiers across four different combinations of biomarkers to generate the risk of malignancy predictions. The ML classifiers included: C5.0 rule-Based (C5), decision trees (DT), elastic net (EN), multivariate adaptive regression spline (MARS), naïve Bayes (NB), neural net (NN), polynomial supervised vector machine (SVM), RF, and extreme gradient boosted trees (XGBoost). ML classifiers were trained across the 25 bootstrap resamples. When computationally feasible, each model’s hyperparameters were tuned using Latin hypercube sampling with a grid size of 50. The NN was a single layer, feed-forward NN, trained using 2 hidden units, a dropout rate of 0.3, and 100 epochs. Receiver operator characteristic (ROC) curves, ROC AUC values, and accuracy were calculated for each hyperparameter combination across each bootstrap resample.

The ML classifiers were then compiled into an ensemble stack using the least absolute shrinkage and selection operator (LASSO) across six distinct penalty values. Penalty, also known as regularization, causes coefficient estimates with minor contributions to be eliminated from the model. The final ensemble stack models, and each of the individual classifiers were then applied to the testing dataset to make final predictions. For each predictive algorithm, the ROC AUC, accuracy, sensitivity, specificity, NPV, and positive predictive value (PPV) was calculated. PPV and NPV were calculated based on the prevalence within this dataset. Using 10,000 bootstrap resamples and DeLong’s non-parametric approach, the ROC AUC values of the four final ensemble models were compared [17,18].

### 2.6. Software

All statistical analysis was performed with R programming language v.4.0.3 [19]. Packages used were ‘*tidymodels*’ v.0.1.2, ‘*tidyverse*’ v.1.3.0, ‘*stacks*’ v.0.1.0, ‘*workflowsets*’ v.0.2.3, ‘*pROC*’ v.1.17.0.1, ‘*discrim*’ v.1.1, ‘*rules*’ v.0.1.0, ‘*readxl*’ v.1.3.1, ‘*RColorBrewer*’ v.1.1-2, ‘*viridis*’ 0.5.1, ‘*GGally*’ v.2.0.0, and ‘*corrplot*’ v.0.84 [20]. The code is available at: https://github.com/reid-inveen-shaw/ovca_ml (uploaded on 20 February 2022).

## 3. Results:

### 3.1. Patient Demographic, Univariate Statistical Analysis, and Logistic Regression

A total of 140 patients with a pelvic mass were included in this study. Study participants had an average age of 54.9 years with a range from 20 to 91 years (Table 1). A total of 91.4% of patients were white and 64.3% were post-menopausal. None of the variables were missing at a rate of more than 15%. Menopausal status was collected for every patient. A total of 43.5% of patients were diagnosed with a malignancy, including epithelial ovarian cancer (EOC), non-epithelial ovarian cancer, metastatic cancer, borderline tumors, and other gynecologic cancers (Table S2). The remaining 79 women were diagnosed with a benign tumor. Benign tumors consisted of cystadenomas, endometriomas, mature teratomas, and others (Table S2).

Statistical significance of differences of cancer versus benign groups was determined. Twenty-five biomarkers showed a statistically significant (false discovery rate corrected *p*-value < 0.01) difference in malignant versus benign samples (Figure S1). AGP1, CA125, CRP, Cyfra21.1, FER, FETA, FOLR1, HE4, LRG1, NCAM, TRF, and YKL40 were significantly differentially present in plasma, and AAT, AGP1, ALCAM, C4, CEACAM1, FER, HE4, ITIH4, LRG1, NCAM, and VCAM in urine. AGP1, FER, HE4, LRG1, and NCAM differed significantly between cases in controls when measured in both plasma and urine. 

Upon fitting a LR model with all 25 statistically significant variables and iteratively eliminating each variable, average model performance (ROC AUC) decreased most significantly with the elimination of LGR1 (plasma), AGP1 (plasma), and AGP1 (urine) (Figure S2A). Elimination of architect-measured CA125 resulted in the largest decrease in specificity, followed closely by bead-based NCAM (plasma), CRP (plasma), RBP4 (urine), and architect-measured HE4 (Figure S2B). Sensitivity decreased most with the elimination of AGP1 (plasma), SVCAM (urine), and YLK40 (Figure S2B). 

### 3.2. Feature Selection through Orthogonal Approaches

**Figure 1:**
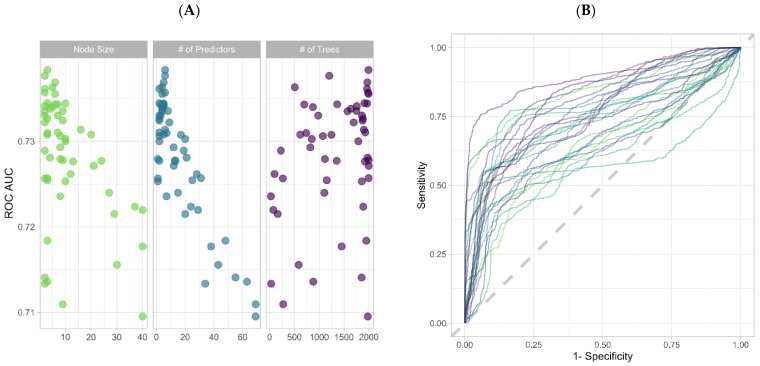

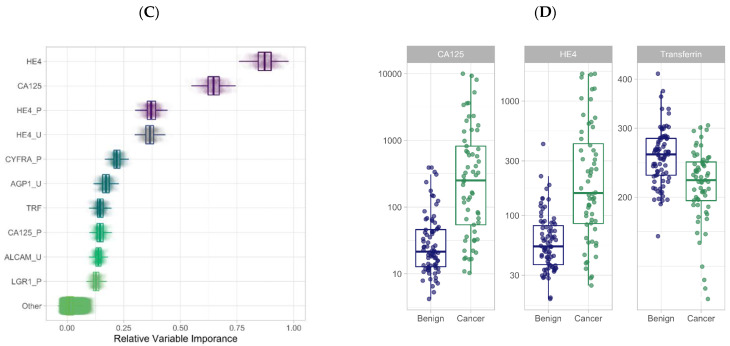
(**A**) Dot plot of ROC AUC for each of the three tuned random forest (RF) hyperparameters using Bayes optimization. (**B**) ROC curves of the RF hyperparameters averaged across each of the 25 bootstrap resamples. (**C**) Boxplot of variable importance as calculated by Gini impurity across 1000 permutations of the RF model fitting. “P” indicates plasma, whereas “U” indicates urine; unlabeled biomarkers are from serum. (**D**) Boxplot of the three selected predictive parameters: CA125, HE4, and transferrin.

Using Bayesian optimization to tune the hyperparameters of the embedded RF model, we calculated 82,800 unique predictions. Model performance increased when the minimal node size and the number of randomly selected variables were low, while the number of trees was large (Figure 1A,B). The top 10% of unique predictive parameters as identified by Gini impurity were: HE4, CA125, Cytokeratin 19 Fragment (plasma), AGP1 (urine), TRF (serum), ALCAM (urine), and LRG1 (plasma) (Figure 1C). HE4 from serum, urine, and plasma were three of the four analytes with the largest average Gini impurity values (Figure 1C). Each pairwise combination of HE4 measurements showed a statistically significant positive Spearman rank correlation (*p*-value < 0.05) (Figure S3). TRF ranked seventh by way of Gini variable importance and had the largest negative loading score in the PCA (Figure 1C, Figure S4A,B). HE4 and CA125 values were significantly elevated in malignancy, while TRF was significantly decreased in malignancy (Figure 1D). HE4 and CA125 both had a statistically significant negative correlation with TRF by way of the Spearman rank coefficient (Figure S3). Four combinations of predictive parameters were chosen for ensemble stack training: HE4 and CA125; HE4, CA125, and TRF; top 10% of parameters by Gini impurity; and all predictive parameters.

### 3.3. Training ML Classifiers and Generating an Ensemble Stack Using LASSO Regression

**Figure 2:**
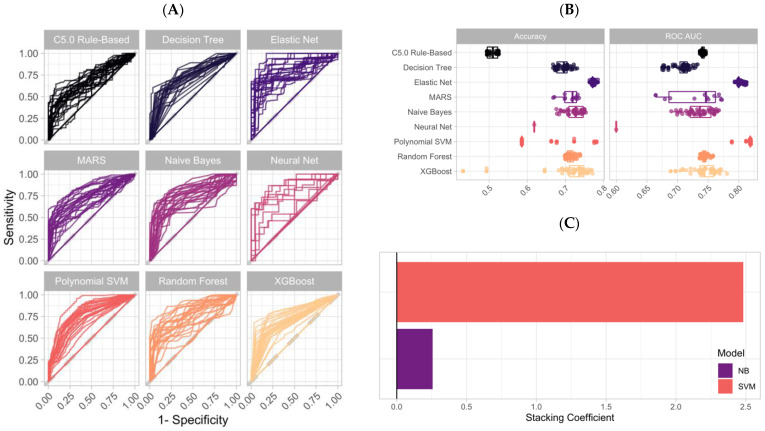
(**A**) 25 ROC curves for each of the nine unique ML classifiers. One curve represents the average of all tested hyperparameters for one of the bootstrap resamples. (**B**) ROC AUC values for each of the individual ML hyperparameters averaged across each of the 25 bootstrap resamples. (**C**) Ensemble stack ML classifier weights as defined by LASSO regression.

All four ensemble stacks underwent the same training methods, but for the sake of clarity and brevity, only the training results of the three-marker ensemble stack will be reported. The ensemble stack of HE4, CA125, and TRF was generated from 314 unique ML classifiers and 392,247 predictions on the training dataset (Figure 2A, Figure S5A–H). The largest ROC AUC averaged across all the bootstrap resamples for each of the classifiers was 0.74 with C5, 0.81 with EN, 0.78 with MARS, 0.77 with NB, 0.68 with NN, 0.76 with RF, 0.82 with SVM, 0.73 with DT, and 0.78 with XGBoost (Figure 2B). The highest accuracy among each model was 52.5% with C5, 78.5% with EN, 73.6% with MARS, 75.3% with NB, 61.2% with NN, 73.9% with RF, 78.1% with SVM, 73.1% with DT, and 77.1% with XGBoost (Figure 2B). The 314 ML classifiers were then compiled into one ensemble stack using LASSO regression across six distinct penalty values (Figure 2C). NB, and SVM were selected as final ensemble stack components, with SVM having the largest coefficient weight (Figure 2C). For each of the three other ensemble stacks, approximately 400,000 malignancy predictions were calculated during model training. A final LASSO penalty of 0.1 was used for all models; however, the individual algorithm components were unique (Figure S6A–D).

### 3.4. Application of an Ensemble Stack Generates Accurate Predictions on the Testing Dataset

**Figure 3:**
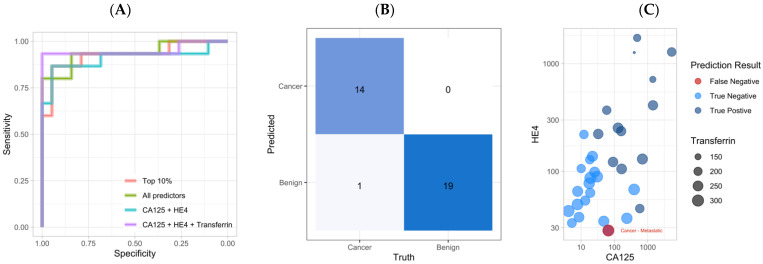
(**A**) ROC curves of the four ensemble stack models. No statistical difference was observed using DeLong’s method. (**B**) Ensemble stack confusion matrix for the final testing data (*n* = 34). (**C**) Dot plot of the testing samples colored by prediction result. CA125 is on the *X*-axis, HE4 is on the *Y*-axis, and transferrin is illustrated by the size of each dot.

When all 70 predictors were included for model generation, the final ensemble stack consisted of two EN and two MARS classifiers (Figure S6A). The ROC AUC on the testing dataset was 0.909 (Table 2). The final members of the ensemble stack using the top 10% (*n* = 7) predictors included two EN, one MARS, and three NB classifiers (Figure S6B). The ROC AUC was 0.926 (Table 2). The ensemble stack using HE4 and CA125 included one EN, one MARS, and one NB classifier with a final ROC AUC of 0.937 (Figure S5C, Table 2). The ensemble stack of HE4, CA125, and TRF generated the largest ROC AUC at 0.951 (Table 2). No pairwise statistical difference was observed between the ROC AUC of any of the four ensemble stack models when assessed by 10,000 bootstrap resamples or DeLong’s method (*p*-value > 0.05) (Figure 3A) [17,21].

The tri-variable ensemble stack demonstrated an accuracy of 97.1%, and a sensitivity of 93.3% with a specificity of 100% (Table 2). The training ROC AUC of all the individual classifiers is below that of the testing AUC, ensuring that no overfitting of the models occurred. The final model outperformed each of the individual classifiers with respect to accuracy, specificity, PPV, and NPV (Table S3). The ensemble stack and the individual SVM classifier had the same sensitivity, and ROC AUC value (Table S3). The tri-variable ensemble made one incorrect prediction: a false negative classification of a poorly differentiated high-grade metastatic tumor suspected to be of upper gastrointestinal (GI) origin (Figure 3C). All the remaining samples were correctly predicted within the testing dataset: a borderline tumor (*n* = 1), benign tumors (*n* = 19), early-stage (stage I or II) EOC (*n* = 4), and late-stage (stage III or IV) EOC (*n* = 9) (Figure 3C).

## 4. Discussion

The two aims of this study were to identify essential markers of a malignant pelvic mass and develop an accurate predictive algorithm of disease. This study evaluates four unique combinations of biomarkers from a panel of 70 parameters, combining hundreds of ML models into an ensemble stack with LASSO regression. One recent study utilized ML algorithms to make risk assessments of EOC vs. benign tumors using a panel of non-specific biomarkers [22]. However, we included all types of malignant pelvic masses into our predictive algorithm and investigated a large panel of cancer-specific markers. While grouping EOC with non-EOC, other gynecologic cancers, borderline tumors, and metastatic cancers may pose a significant challenge to accurate prediction, it has the potential to be more beneficial for patients and improve clinical outcomes. 

To identify the best combination of predictive parameters of disease, prevent model overfitting, and reduce computational requirements, we performed feature selection of all of the unique measurements and generated four distinct ensemble stacks [23]. We used an orthogonal approach of Gini impurity calculations within a RF classifier, and principal component analysis to identify important predictive parameters. Patient age, which has been demonstrated to be the greatest independent risk factor for EOC, did not provide any additional predictive benefit to the model [24]. Menopausal status, which is incorporated into many different predictive algorithms, did not improve model performance. While not statistically significant from the combination of HE4 and CA125, the combination of HE4, CA125, and transferrin had the greatest ROC AUC, sensitivity, and specificity compared to the other ensemble stack algorithms. HE4 and CA125 are routinely measured analytes in determining the preoperative risk of an ovarian mass to be malignant. HE4, which is known to be inversely correlated with kidney function, was not adjusted for within the dataset [25]. Transferrin, a glycoprotein commonly known for maintaining iron homeostasis and as a negative acute-phase reactant, is less commonly assessed, but used in OVA1^®^ and OVERA^®^. Although model performance decreased with more than three parameters, this finding may change with increasing the training dataset sample size or incorporating different variables.

The combination of a highly sensitive, yet specific set of biomarkers with a sophisticated ML algorithm is of significant clinical utility. A high NPV allows patients with a benign tumor to remain with their gynecologist and in their community for surgery without the risk of being diagnosed with a malignancy. For those patients with an asymptomatic complex ovarian cyst, they may be able to avoid invasive, and costly surgeries. Similarly, a test with a high PPV allows for timely and necessary referrals of patients with a malignant diagnosis to specialists and institutions experienced in the management of ovarian cancer. Improved triage may have a positive effect on morbidity and mortality, as high-volume institutions have continually demonstrated better outcomes and greater rates of survival for patients with an ovarian malignancy [1,2,3].

Of the 34 samples within the testing dataset, the final tri-variable ensemble model made one incorrect prediction, whereas the two-variable model made five incorrect predictions. The one incorrect prediction of the tri-variable model was a metastatic cancer, likely of GI origin, predicted to be benign. This prediction is not completely unsurprising as none of the biomarkers used in our model are known to be strong predictors of GI cancer [26,27]. Equally, the prognosis for this patient was not changed as the stage was advanced at the time of testing and diagnosis. Importantly, the model correctly identified all patients with an early stage 1 and 2 EOC, which are associated with higher rates of survival [5]. With an increased sample size of malignant diagnoses, future work would be aimed to predict the primary site of disease and possibly the histological subtype of disease. This would likely require the addition of at least one more biomarker and the creation of a multiclass classification model.

This is the first time that an ensemble stack has been used to predict the risk of malignancy of a pelvic mass. This method is powerful, as it blends a heterogeneous group of algorithms to expose distinct, yet complementing aspects of the data [28]. Furthermore, this method may avoid model overfitting, while limiting the bias and variance that is often associated with ML [29,30]. EN, MARS, NB, and SVM, the four classifiers identified as members of the final ensemble stack algorithms, have been previously shown to be effective classification algorithms [31,32,33,34]. Likely due to the limited number of continuous predictive parameters, no tree-based classifiers were included in any of the final ensemble stacks [35]. Similarly, NN, which improves in performance with large numbers of observations and data points, was not included in any of the final models.

Limitations of this study are with respect to the sample size and patient demographics. An increase in the number of patient samples will likely improve ML model performance, as we can train our model over edge cases. Another limitation of the study is only 6.5% of women diagnosed with a malignancy identified as a race other than white. However, closer to 77% of women with an ovarian cancer diagnosis are not white [5]. Unfortunately, the bias within this dataset is not limited to this study and improvements to this model and the applicability to broader populations require a more diverse and representative original patient population [36]. Furthermore, 44% of the ovarian tumors within this dataset were diagnosed as malignant, but the true prevalence of malignancy after the identification of a pelvic mass is reported to be closer to 10–20% [4]. These differences may lead to inaccurate model generation as some ML models incorporate prevalence in model training. Due to the limited sample size and possibility of inappropriate redundancy, we did not perform oversampling of benign samples or under sampling of malignant samples.

The study demonstrates a superior performance of optimized clinical assays for CA125 and HE4 vs. research grade tests, indicating that assays for other individual promising biomarkers could be further optimized for a superior tri-biomarker classifier. However, to identify promising individual biomarkers that complement CA125/HE4 algorithm, the study needs to be performed on a much higher number of individuals to create reliable and statistically significant data. Our data demonstrate that multiplexing does not affect the performance as individual ELISA assay for YKL40 performed similarly as multiplex bead-based assay. We also observed a more robust performance of some biomarkers when measured in blood vs. urine but analyzing two different bodily fluids is more expensive and labor intensive; thus, a common test using a single fluid (blood) is preferable.

## 5. Conclusions

Combining serum levels of CA125, HE4, and transferrin with the stacking of multiple ML classifiers into an ensemble can provide valuable preoperative diagnostic predictions for women diagnosed with a pelvic mass. Prospective trials with cancer incidence rates similar to the general population of women with a pelvic mass are need in order to determine if transferrin has added value to the dual marker combination of HE4 and CA125.

## Figures and Tables

**Table 1:** Patient demographics and tumor characteristics.

**A. Patient demographics**					
	Training (*n* = 106)		Testing (*n* = 34)	All Patients (*n* = 140)	
Age					
Years (range)	54.7 (20–91)		55.3 (25–82)	54.9 (20–91)	
Race					
Black	2 (1.9%)		1 (2.9%)	3 (2.1%)	
Hispanic	1 (0.9%)		1 (2.9%)	2 (1.4%)	
Other	6 (5.7%)		1 (2.9%)	7 (5.0%)	
White	97 (91.5%)		31 (91.2%)	128 (91.4%)	
Menopausal Status					
Post-menopausal	68 (64.2%)		22 (64.7%)	90 (64.3%)	
Pre-menopausal	38 (35.8%)		12 (35.3%)	50 (35.7%)	
Histological Diagnosis					
Benign	60 (56.6%)		19 (55.9%)	79 (56.4%)	
Borderline/LMP	3 (2.8%)		1 (2.9%)	4 (2.9%)	
Cancer—EOC I–II	17 (16.0%)		4 (11.8%)	21 (15.0%)	
Cancer—EOC III–IV	15 (14.2%)		9 (26.5%)	24 (17.1%)	
Cancer—Metastatic	5 (4.7%)		1 (2.9%)	6 (4.3%)	
Cancer—EOC Unstaged	1 (0.9%)		0 (0.0%)	1 (0.7%)	
Cancer—Non-EOC	2 (1.9%)		0 (0.0%)	2 (1.4%)	
Cancer—Other Gyn	3 (2.8%)		0 (0.0%)	3 (2.1%)	
Stage					
IA	8 (7.5%)		2 (5.9%)	10 (7.1%)	
IB	1 (0.9%)		0 (0.0%)	1 (0.7%)	
IC	6 (5.7%)		1 (2.9%)	7 (5.0%)	
II	1 (0.9%)		0 (0.0%)	1 (0.7%)	
IIA	3 (2.8%)		0 (0.0%)	3 (2.1%)	
IIB	1 (0.9%)		0 (0.0%)	1 (0.7%)	
IIC	1 (0.9%)		1 (2.9%)	2 (1.4%)	
IIIA	5 (4.7%)		2 (5.9%)	7 (5.0%)	
IIIB	1 (0.9%)		1 (2.9%)	2 (1.4%)	
IIIC	12 (11.3%)		7 (20.6%)	19 (13.6%)	
IV	5 (4.7%)		1 (2.9%)	6 (4.3%)	
Unstaged	1 (0.9%)		0 (0.0%)	1 (0.7%)	
**B. Malignant tumor histology**					
EOC Type	Stage I	Stage II	Stage III	Unstaged	All
Clear Cell	2 (33.3%)	1 (16.7%)	3 (50.0%)	0 (0.0%)	6 (13.0%)
Endometrioid	1 (50.0%)	0 (0.0%)	1 (50.0%)	0 (0.0%)	2 (4.3%)
Mucinous	7 (87.5%)	1 (12.5%)	0 (0.0%)	0 (0.0%)	8 (17.4%)
Serous	4 (13.8%)	4 (13.8%)	20 (69.0%)	1 (3.4%)	29 (63.0%)
Mixed	0 (0.0%)	1 (100%)	0 (0.0%)	0 (0.0%)	1 (2.2%)
All EOC	14 (30.4%)	7 (15.2%)	24 (52.2%)	1 (2.2%)	46 (100%)
**C. Malignant tumor grade**					
	All patients		Pre-Menopausal	Post-Menopausal	
EOC Grade	*n* (%)		*n* (%)	*n* (%)	
Grade 1	9 (19.6%)		3 (30.0%)	6 (16.7%)	
Grade 2	3 (6.5%)		1 (10.0%)	2 (5.6%)	
Grade 3	34 (73.9%)		6 (60.0%)	28 (77.8%)	
Total	46 (100%)		10 (21.7%)	36 (78.3%)	

**Table 2:** Ensemble model statistics on testing dataset.

Ensemble Model	AUC	Accuracy	Sensitivity	Specificity	PPV	NPV
CA125 + HE4 + TRF	0.951	97.1%	93.3%	100.0%	100.0%	95.0%
CA125 + HE4	0.937	85.3%	86.7%	84.2%	81.2%	88.9%
Top 10%	0.926	82.4%	66.7%	94.7%	90.9%	78.3%
All predictors	0.909	88.2%	80.0%	94.7%	92.3%	85.7%

## Data Availability

The code is available at: https://github.com/reid-inveen-shaw/ovca_ml (uploaded on 20 February 2022).

## Supplementary Materials

The following supporting information can be downloaded at: https://www.mdpi.com/article/10.3390/cancers14051291/s1, Figure S1. Univariate analysis of non-parametric Mann-Whitney test. Figure S2. Logistic Regression model of 25 statistically significant variables. Figure S3. Pairwise Spearman rank coefficient of all numerical variables. Figure S4. (A) Principal Component Analysis of all numerical variables. (B) First principal component (*X*-axis) plotted against the second principal component (*Y*-axis), beginning to separate the benign from cancer samples. Figure S5. Model-specific hyperparameter tuning results of the three-variable biomarker panel across the training dataset. Figure S6. Lasso Regression results of the four ensemble stacks. Table S1. Demographic and biomarker variables. Table S2. Histopathology of benign and malignant tumors. Table S3. Tri-variable ensemble stack statistics from the testing dataset.
