# Supplementary material for: Stacking Machine Learning Algorithms for Biomarker-Based Preoperative Diagnosis of a Pelvic Mass
Source: Cancers (Basel). 2022 Mar 2;14(5):1291. doi: 10.3390/cancers14051291 (PMC8909341; doi:10.3390/cancers14051291)
Supplement: Supplementary file 1 [file cancers-14-01291-s001.zip › cancers-1561524-supplementary.pdf]

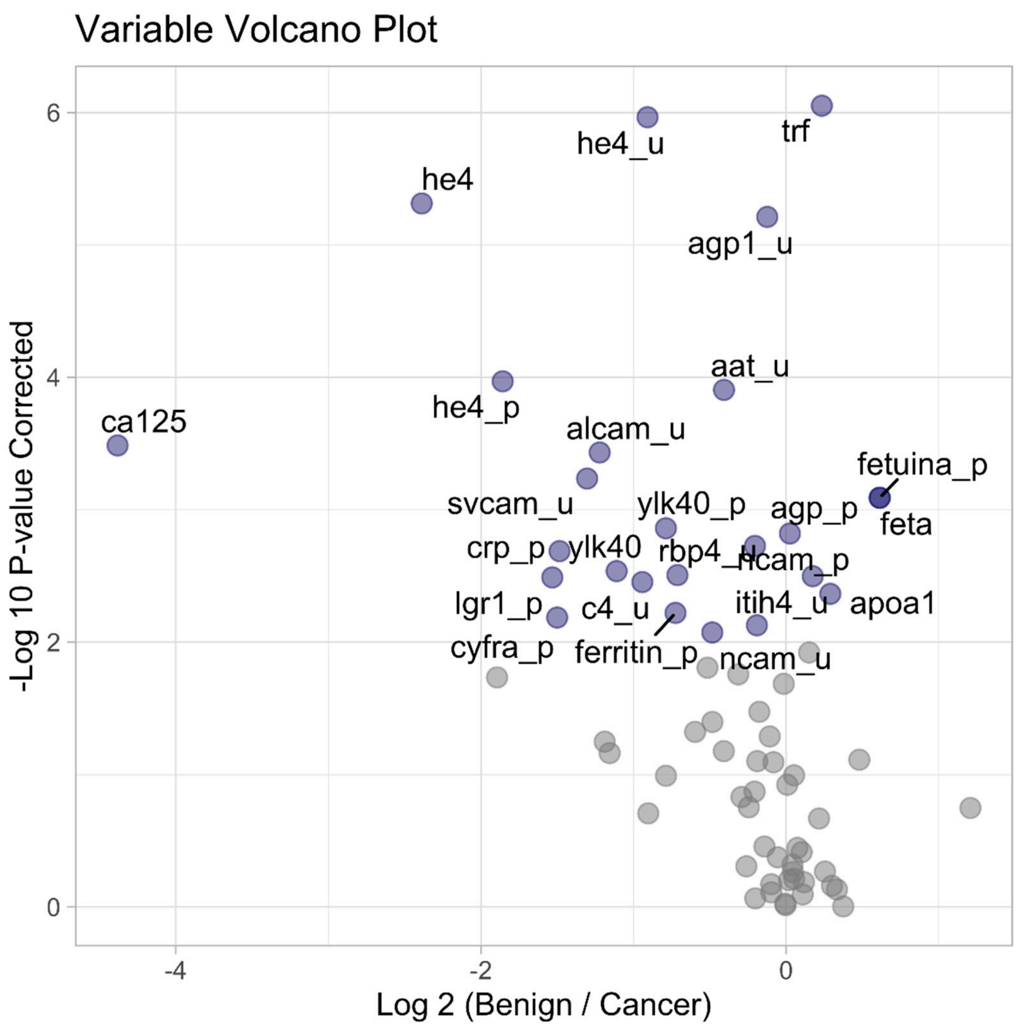

Figure S1. Univariate analysis of non-parametric Mann-Whitney test. *p*-values are corrected with False Discovery Rate (FDR) correction. Biomarkers with corrected *p*-value < 0.01 are labeled.

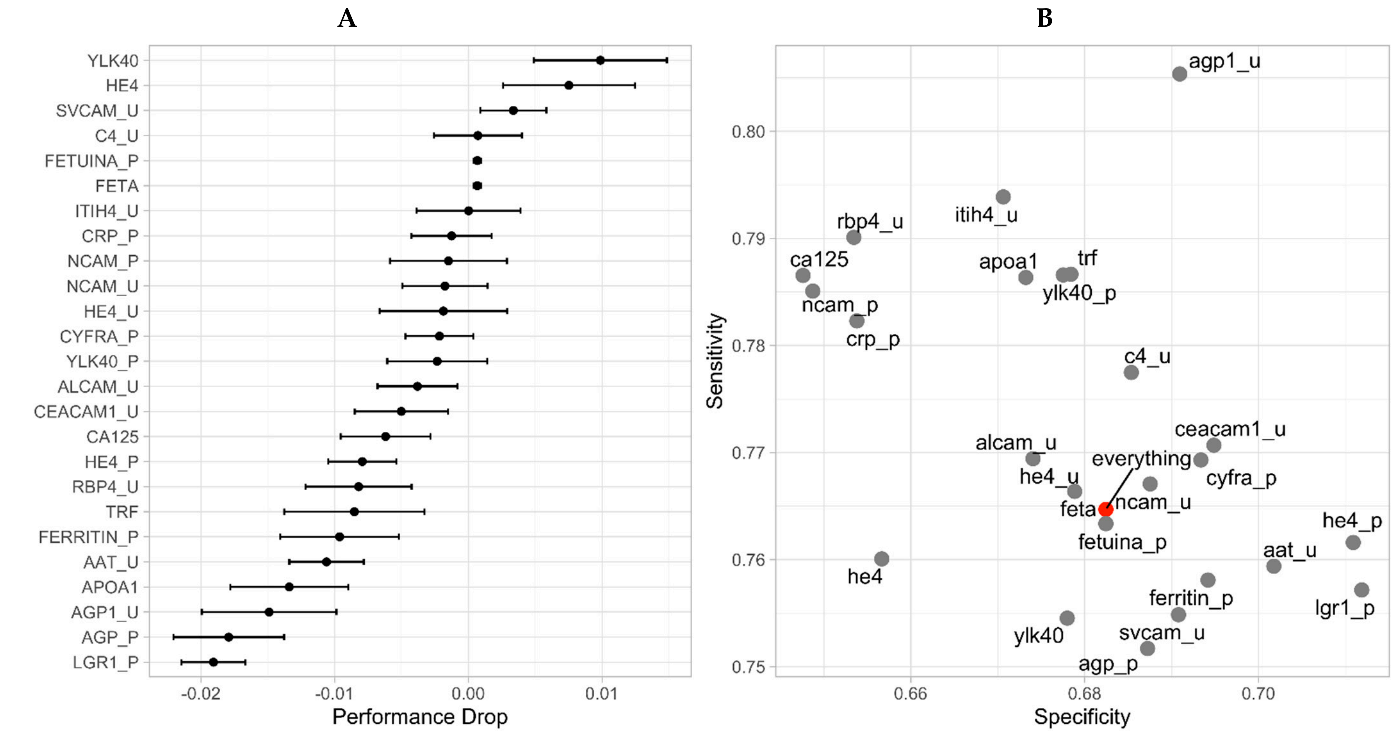

Figure S2. Logistic Regression model of 25 statistically significant variables. (**A**) Positive values indicate improved model performance (ROC AUC) with biomarker exclusion from the logistic regression model. The mean performance drop is represented by the circle; standard error is indicated by the error bars. (**B**) Specificity versus sensitivity dot plot. Labels indicate when that biomarker is excluded from the logistic regression model.

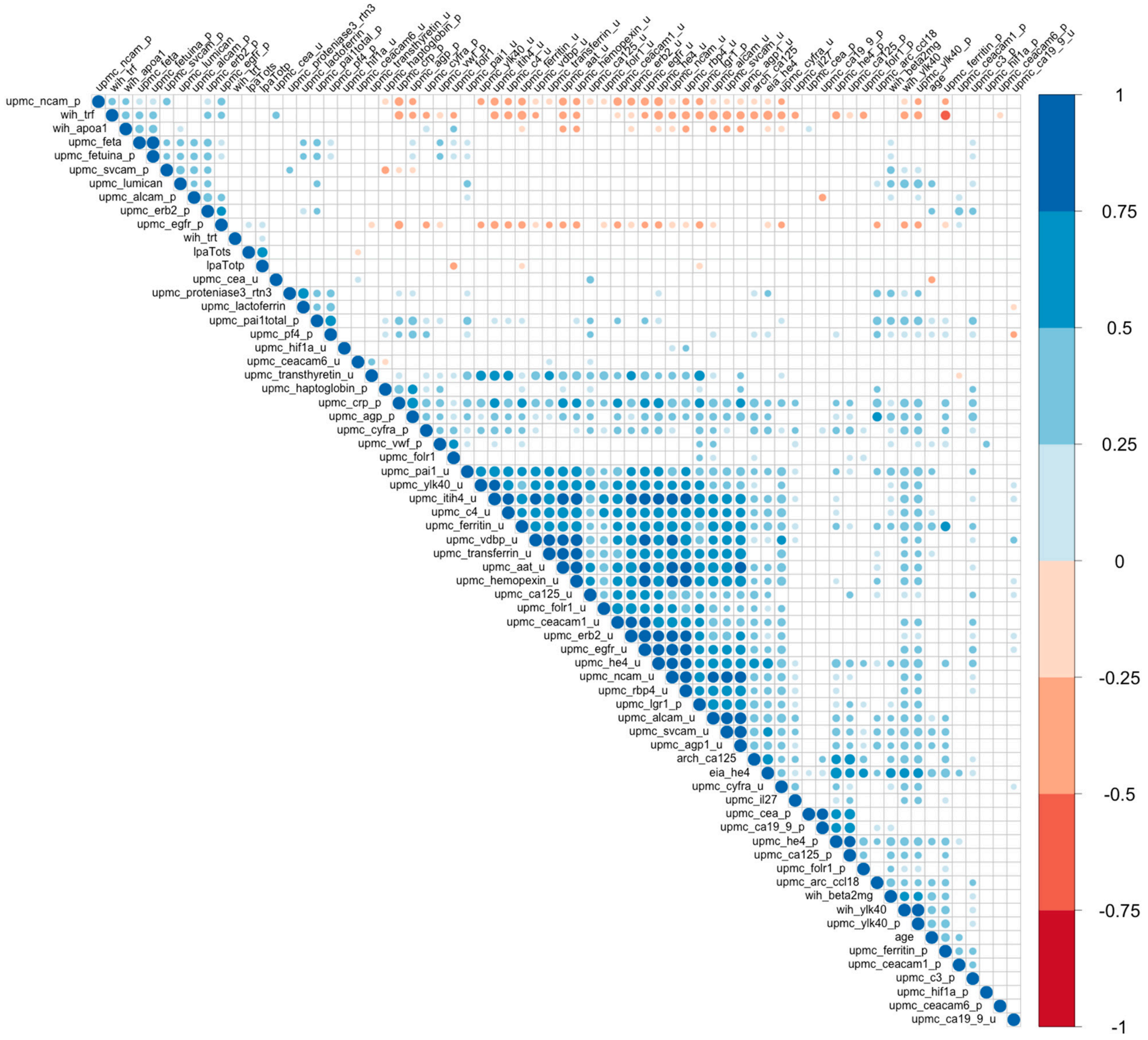

Figure S3. Pairwise Spearman rank coefficient of all numerical variables. Non-significant correlations are left white. Negative correlations are indicated in red. Positive correlations are indicated in blue.

A

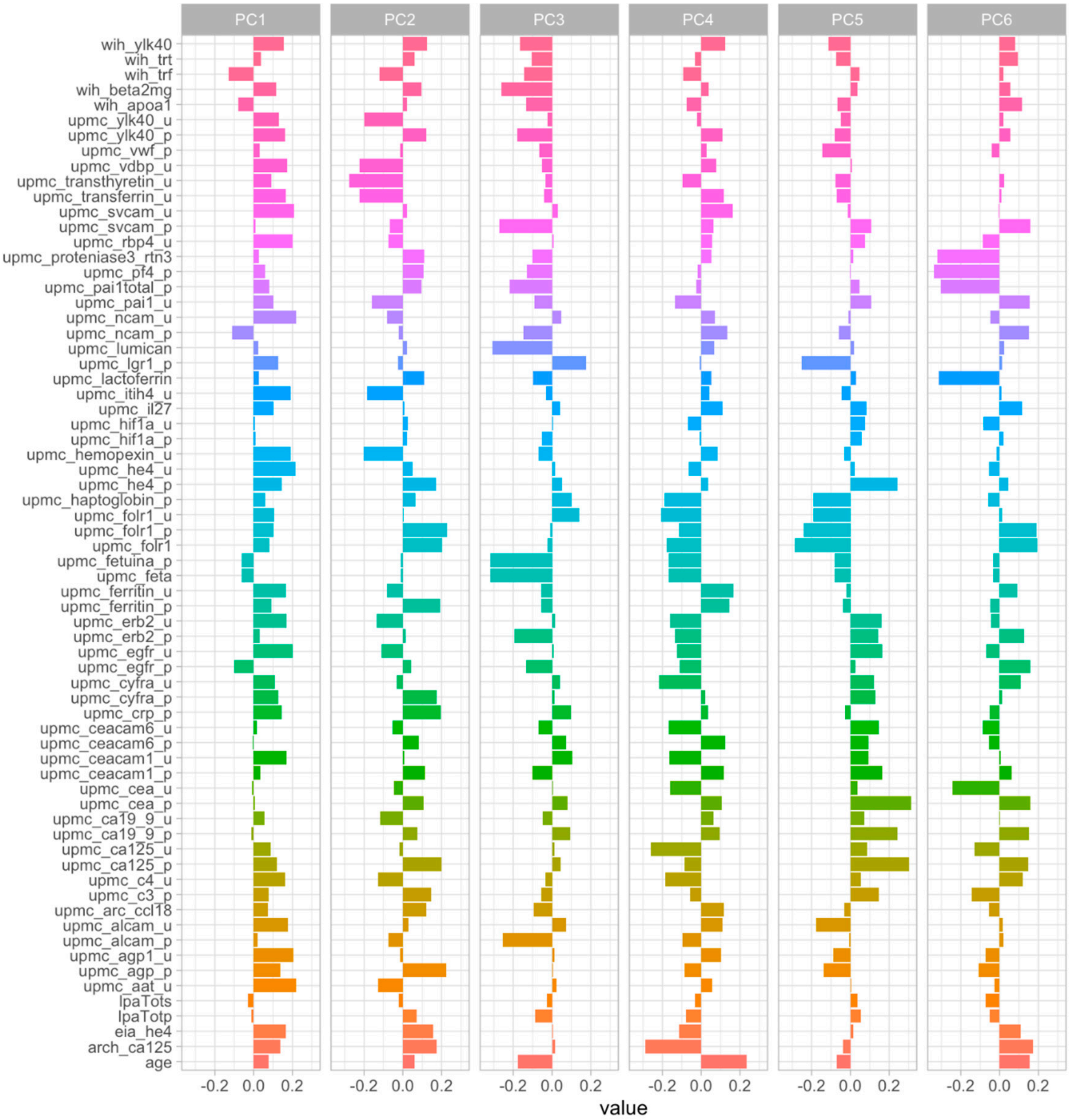

B

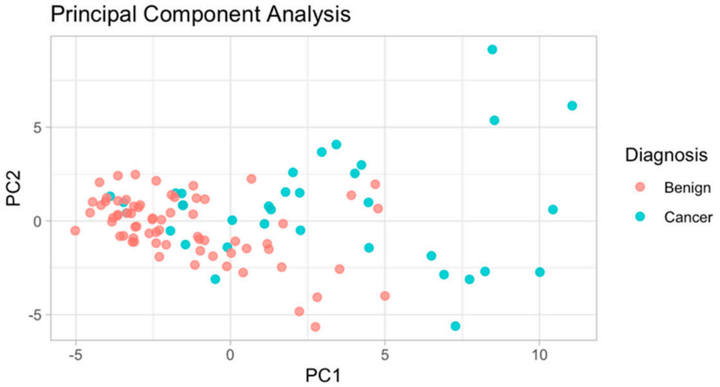

Figure S4. (**A**) Principal Component Analysis of all numerical variables. (**B**) First principal component (x-axis) plotted against the second principal component (y-axis), beginning to separate the benign from cancer samples.

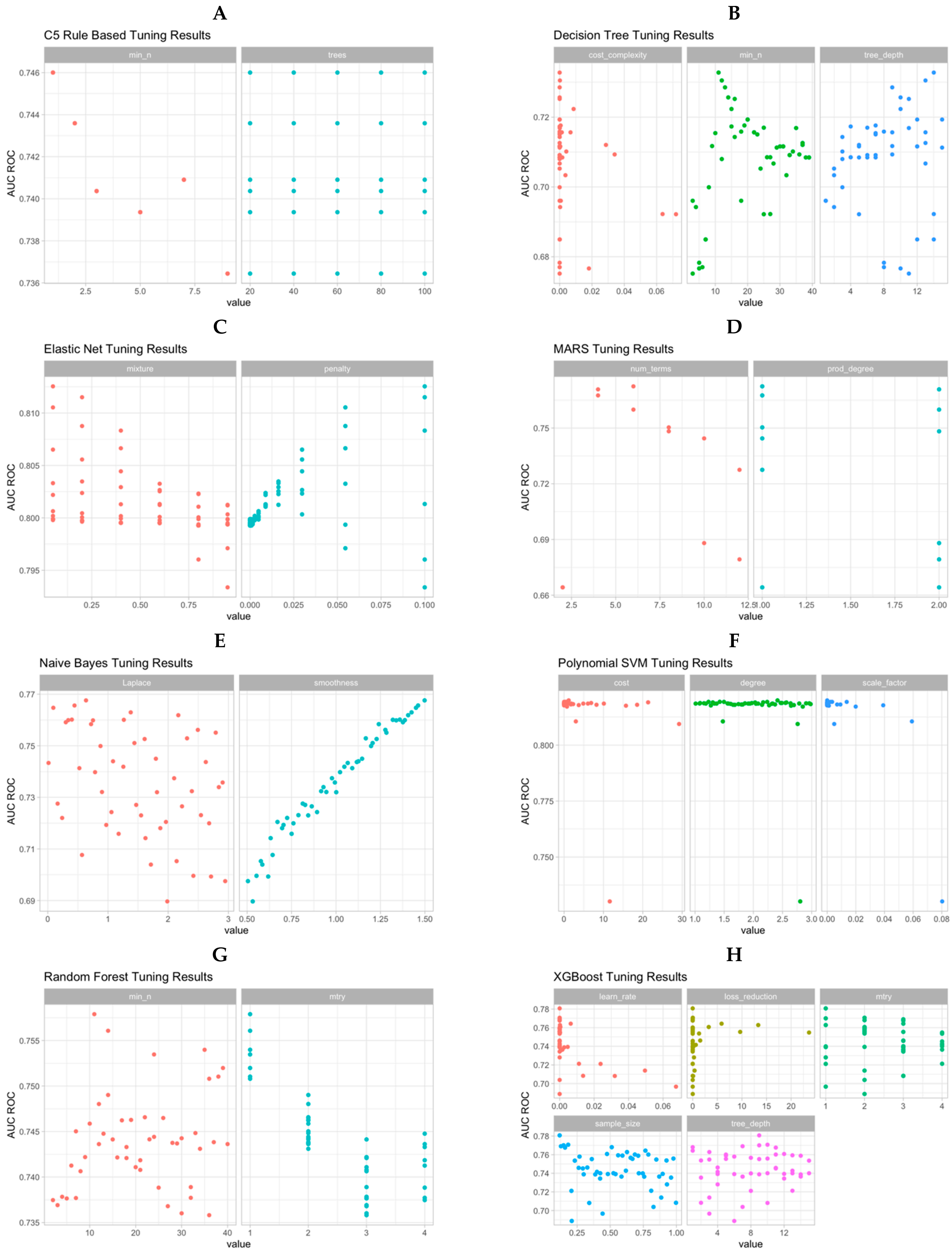

Figure S5. Model-specific hyperparameter tuning results of the three-variable biomarker panel across the training dataset. (**A**) C5.0 Rule-based; (**B**) Decision Tree; (**C**) Elastic Net; (**D**) Multivariate regression spline (MARS); (**E**) Naïve Bayes; (**F**) Polynomial Support Vector Machine; (**G**) Random Forest; (**H**) Extreme Gradient Boosted Trees (XGBoost). The Neural Net classifier was not tuned across a grid of parameters.

A

B

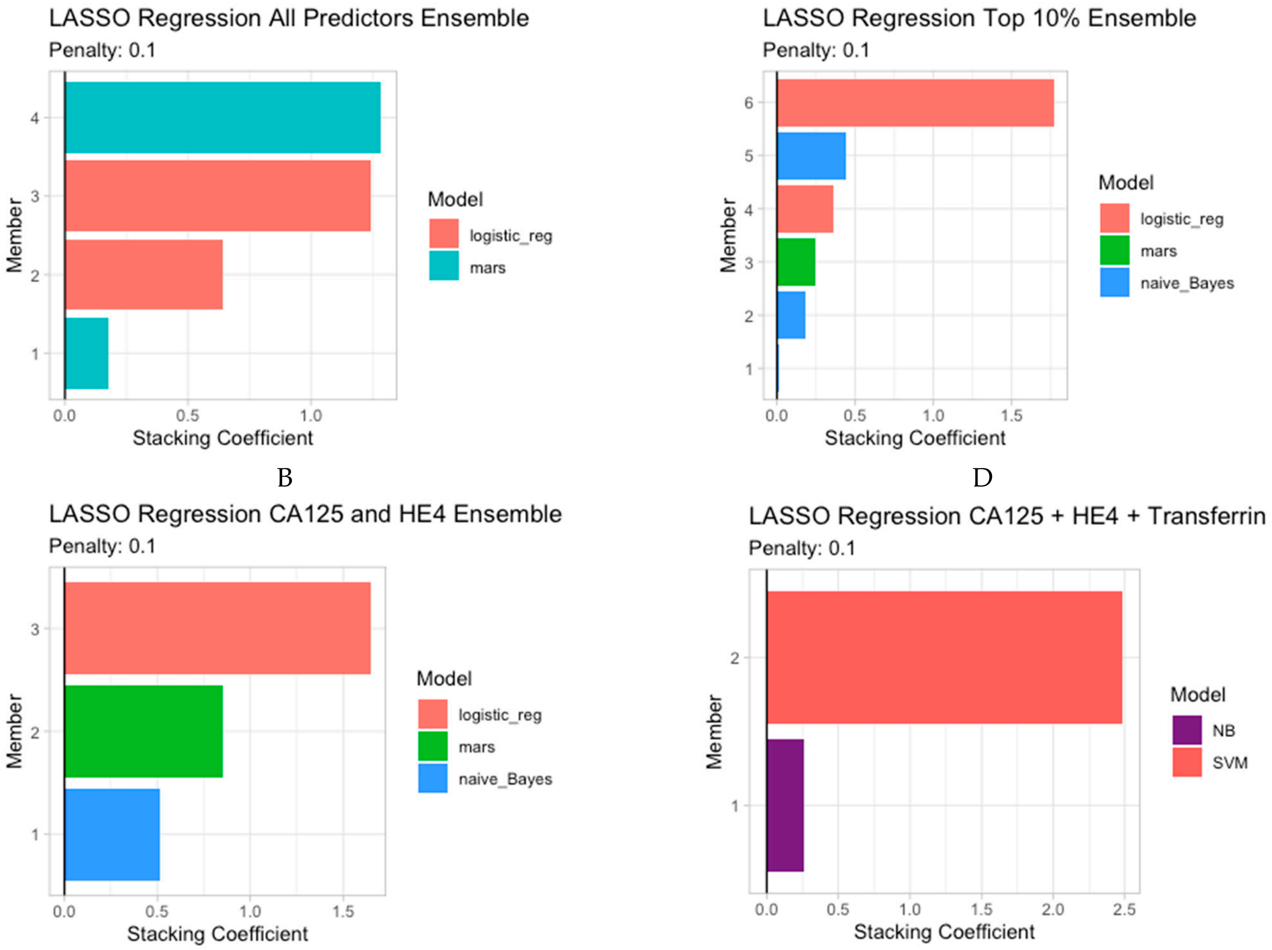

Figure S6. Lasso Regression results of the four ensemble stacks. (**A**) All predictors. (**B**) Top10% of predictors as defined by largest Gini impurity. (**C**) CA125 + HE4. (**D**) CA125 + HE4 + Transferrin.

Table S1. Demographic and biomarker variables.

**Variables**	
Age	CEACAM-1 (U)
Menopausal status	CEACAM-6 (U)
CA125	Ferritin (U)
HE4	NCAM (U)
YKL-40	sVCAM (U)
Transthyretin	PAI-1 (U)
ApoA1	YKL40 (U)
Beta2 Microglobulin	FOLR1 (U)
Transferrin	FETA
LPA (S)	IL-27
LPA (P)	Lumican
HE4 (P)	ARC/CCL18
Cyfra (P)	Proteinase 3/RTN3
CA125 (P)	CRP (P)
CEA (P)	Fetuin A (P)
ErbB2 (P)	AGP (P)
EGFR (P)	Haptoglobin (P)
CA 19-9 (P)	PF4 (P)
HIF-1a (P)	vWF (P)
ALCAM (P)	C3 (P)

CEACAM-1 (P)	FOLR1
CEACAM-6 (P)	LRG1 (U)
Ferritin (P)	ITIH4 (U)
NCAM (P)	C4 (U)
sVCAM (P)	AAT (U)
PAI-1 (total) (P)	AGP-1 (U)
YKL40 (P)	Hemopexin (U)
FOLR1 (P)	RBP-4 (U)
HE4 (U)	Transferrin (U)
Cyfra (U)	Transthyretin (U)
CA125 (U)	VDBP (U)
CEA (U)	Lactoferrin
ErbB2 (U)	ALCAM (U)
EGFR (U)	CEACAM-1 (U)
CA 19-9 (U)	CEACAM-6 (U)
HIF-1a (U)	Ferritin (U)
ALCAM (U)	

Table S2. Histopathology of benign and malignant tumors.

**Malignancies**	
Epithelial Ovarian Cancer	
Serous	28
Endometrioid	2
Mucinous	8
Clear Cell	6
Mixed Serous and Endometrioid	2
Germ Cell Tumor	2
LMP Serous	2
LMP Mucinous	2
Uterine Malignancy	3
Metastatic to the Ovary	6
**Benign Tumors**	
Serous Cystadenoma and Cystadenofibroma	20
Mucinous	16
Endometriosis	13
Teratoma	9
Fibroma / Fibrothecoma	7
Brenner Tumor	0
Simple / Hemorrhagic Cysts	11
Leiomyoma	1
Endometrial Polyp	1
Endometrial Hyperplasia	1
**Total Number of Malignant and Benign Tumors**	**140**

Table S3. Tri-variable ensemble stack statistics from the testing dataset.

Model	AUC	Accuracy	Sensitivity	Specificity	PPV	NPV
Ensemble	0.951	97.1%	93.3%	100.0%	100.0%	95.0%
SVM	0.951	94.1%	93.3%	94.7%	93.3%	94.7%
NB	0.867	82.4%	73.3%	89.5%	84.6%	81.0%
